# Impact of Hypoxia on Neutrophil Degranulation and Inflammatory Response in Alpha-1 Antitrypsin Deficiency Patients

**DOI:** 10.3390/antiox13091071

**Published:** 2024-09-02

**Authors:** María Magallón, Silvia Castillo-Corullón, Lucía Bañuls, Teresa Romero, Daniel Pellicer, Alberto Herrejón, María Mercedes Navarro-García, Cruz González, Francisco Dasí

**Affiliations:** 1 Department of Physiology, School of Medicine, University of Valencia, Avda. Blasco Ibáñez, 15, 46010 Valencia, Spain; mamase2@alumni.uv.es (M.M.); lubauls@alumni.uv.es (L.B.); daperoig@alumni.uv.es (D.P.); 2 Instituto de Investigación Sanitaria INCLIVA, Rare Respiratory Diseases Group, Avda. Menéndez y Pelayo, 4, 46010 Valencia, Spain; castillo_sil@gva.es (S.C.-C.); maria.m.navarro@ext.uv.es (M.M.N.-G.); cruz.gonzalez@uv.es (C.G.); 3 Paediatrics Unit, Hospital Clínico Universitario de Valencia, Avda. Blasco Ibáñez, 17, 46010 Valencia, Spain; 4 Department of Paediatrics, School of Medicine, University of Valencia, Avda. Blasco Ibáñez, 15, 46010 Valencia, Spain; 5 Pediatrics Unit, Hospital de Manises, Avda. Generalitat Valenciana, 50, 46940 Manises, Spain; tromero@hospitalmanises.es; 6 Pulmonology Unit, Hospital Universitario Doctor Peset, Avda. Gaspar Aguilar, 90, 46017 Valencia, Spain; herrejon_alb@gva.es; 7 Pulmonology Unit, Hospital Clínico Universitario de Valencia, Avda. Blasco Ibáñez, 17, 46010 Valencia, Spain

**Keywords:** alpha-1 antitrypsin deficiency, COPD, hypoxia, neutrophil, rare respiratory diseases, liver

## Abstract

Background: Alpha-1 antitrypsin deficiency (AATD) is an inflammatory disorder where neutrophils play a key role. Excessive neutrophil activation leads to local hypoxia and tissue damage. Most research on neutrophil function has been conducted under atmospheric conditions (21% O_2_), which may not represent physiological or pathological conditions. This study aimed to determine the effects of hypoxia on neutrophil degranulation and cytokine production in AATD patients. Methods: Neutrophils isolated from 54 AATD patients (31 MZ; 8 SZ; 15 ZZ) and 7 controls (MM) were exposed to hypoxia (1% O_2_) for 4 h. Neutrophil degranulation was assessed by measuring elastase (NE), myeloperoxidase (MPO), lactoferrin, and matrix metalloproteinase-9 (MMP-9) levels using immunoassay-based methods. Pro-inflammatory (IL-8, IL-1 beta, IL-6, and TNF-alpha) and anti-inflammatory (IL-4 and IL-10) cytokine levels were assessed by a Luminex-based method. Results: Our results indicate a significantly increased release of NE (*p* = 0.015), MPO (*p* = 0.042), lactoferrin (*p* = 0.015), and MMP-9 (*p* = 0.001) compared to controls. Pro-inflammatory cytokines show a significant rise in IL-8 (*p* = 0.019), a trend towards increased IL-1 beta (*p* = 0.3196), no change in IL-6 (*p* = 0.7329), and reduced TNF-alpha (*p* = 0.006). Anti-inflammatory cytokines show increased IL-4 (*p* = 0.057) and decreased IL-10 (*p* = 0.05703). Conclusions: Increased neutrophil degranulation and inflammatory phenotype are observed in AATD neutrophils under physiological hypoxia.

## 1. Introduction

Alpha-1 antitrypsin deficiency (AATD) is a rare inherited disease characterized by decreased plasma levels of alpha-1 antitrypsin (AAT), a serine protease inhibitor glycoprotein synthesized and secreted mainly by hepatocytes (and to a lesser extent by monocytes, macrophages, and pulmonary epithelial cells). AAT’s main function is to protect the lungs from damage caused by neutrophils’ proteolytic enzymes, including elastase (NE), cathepsin G, and proteinase 3. The SERPINA1 gene encodes AAT, and although more than 120 mutations have been described at this locus, the most common deficient variants are the S and Z forms (versus the normal M allele). These mutations lead to the misfolding of AAT, which polymerizes and accumulates in the endoplasmic reticulum of hepatocytes (and other producing cells), resulting in chronic inflammation in the liver and leading, in some patients, to the development of severe liver disease (cirrhosis and liver cancer). As a consequence, patients with AATD have low plasma AAT levels, resulting in a reduced ability to inhibit NE and leading to lung parenchymal destruction and the development of chronic obstructive pulmonary disease (COPD). This situation is exacerbated by smoking and repeated and prolonged exposure to dust and smoke in occupational settings and polluting environments. At the pulmonary level, AATD patients’ most frequent clinical manifestation is panacinar emphysema [1].

In addition to the antiprotease activity, AAT has anti-inflammatory and immune-regulatory properties. AATD is increasingly considered an inflammatory disorder in which neutrophils play a key role in the inflammation associated with the pathology. Neutrophil activation increases their capacity to damage nearby tissues through protease, reactive oxygen species (ROS), and pro-inflammatory cytokine release [2,3]. Several studies have shown considerable interaction between the molecular pathways that regulate hypoxia and inflammation, so tissue hypoxia is considered part of the normal inflammatory process. Neutrophils are generated within hypoxic tissue such as the bone marrow, are exposed to intermittent hypoxia in the circulation, and are recruited to sites of infection and inflammation, which are often highly hypoxic. Despite this, most knowledge of neutrophil signaling and function derives from studies performed under atmospheric oxygen conditions (21% O_2_), which do not reflect the physiological or pathological setting in vivo [4,5,6,7,8]. Therefore, this study aimed to investigate the impact of hypoxia on neutrophil degranulation and cytokine profile in AATD patients. We hypothesized that, compared to normal individuals, hypoxia would activate neutrophils in AATD patients.

## 2. Materials and Methods

### 2.1. Study Design

Fifty-four children diagnosed with AATD (31 MZ; 8 SZ; and 15 ZZ) and seven healthy volunteers were recruited from the Pediatrics Units of the Hospital Clínico Universitario Valencia (HCUV), Hospital Dr. Peset (Valencia), and Hospital de Manises (Valencia) from January 2018 to June 2021. The inclusion criteria comprised the following: (1) patients diagnosed with AATD according to the American Thoracic Society/European Respiratory Society recommendations, and (2) control individuals with MM phenotype and no history or clinical findings that suggested a pulmonary or hepatic pathology. Exclusion criteria applicable to both groups were as follows: (1) cardiac dysfunction; (2) active fever or infection; (3) autoimmune diseases; (4) neurological disorders; (5) psychiatric disorders; (6) cancer; (7) treatment with antioxidants three months before sample collection; and (8) surgery less than three months before sampling collection.

### 2.2. Demographic Data and Physical Examination

Anthropometric measurements were obtained from all the participants using standard techniques. Body mass index (BMI) was calculated as kg/m^2^. The serum concentration of AAT was measured by nephelometry, and AAT phenotypes were determined by isoelectrofocusing of serum samples. Pulmonary function was evaluated by spirometry. Liver function was assessed by measuring aspartate aminotransferase (AST), alanine aminotransferase (ALT), and γ-glutamyl transferase (GGT). Normal values are shown in Table 1.

### 2.3. Isolation and Culture of Peripheral Blood Neutrophils

Nine mL of peripheral blood was collected from patients and healthy volunteers after 12 h fasting in 4 mL BD Vacutainer CPT with K2EDTA (Becton Dickinson, Madrid, Spain Cat #362781). Neutrophils were isolated by negative immunomagnetic selection (EasySep™ (#19666; StemCell Technologies, Saint Egreve, France)) according to the manufacturer’s instructions. The purity of isolated neutrophils was verified by flow cytometry using an anti-CD16 b monoclonal antibody. Neutrophils’ purity was, in all cases, higher than 98%. Neutrophil manipulations were performed with extreme care to avoid uncontrolled priming. Purified neutrophils (see Supplementary Materials Figure S1 for further details) were incubated at 9 × 10^5^/mL in RPMI-1640 (#R8758, Sigma-Aldrich, Madrid, Spain) supplemented with 10% inactivated fetal bovine serum (F7524; Sigma-Aldrich), 1% sodium pyruvate (S8636; Sigma-Aldrich), and 1% non-essential amino acids (M7145; Sigma-Aldrich) under hypoxic conditions (1% O_2_) at 37 °C for 4 h as previously described [9]. The culture medium added to the flasks under hypoxic conditions was incubated in the appropriate hypoxic conditions for 3 h before culture to facilitate gas exchange.

### 2.4. Priming and Stimulation of Neutrophils

Neutrophils were primed using the physiological agonist, tumor necrosis factor-alpha (TNF-alpha) (20 ng/mL), for 30 min, followed by the activation with the formylated peptide fMLP (100 nM) for 10 min as previously described [9]. Once the incubations were finished, the supernatants of each culture were centrifuged at 2.000× *g* for 10 min at 4 °C to eliminate possible cell debris. Supernatants were harvested, aliquot, and stored at −80 °C until degranulation and cytokine determinations were performed.

### 2.5. Determination of Neutrophil Degranulation

Levels of elastase, myeloperoxidase (MPO), lactoferrin, and matrix metalloproteinase-9 (MMP-9) were measured in the cell culture supernatants using commercial ELISA kits according to the manufacturer’s instructions. Elastase activity was measured using the Neutrophil Elastase Activity Assay Kit (#600610; Cayman Chemical, Ann Arbor, MI, USA); MPO release was evaluated using the EnzChek^®^ Myeloperoxidase (MPO) Activity Assay Kit (#E33856; Invitrogen, Carlsbad, CA, USA); Lactoferrin using the Human Lactoferrin ELISA Kit (#ab200015; Abcam, Cambridge, UK); and MMP-9 using the MMP-9 Human ELISA Kit (#BMS2016-2; ThermoFisher Scientific, Waltham, MA, USA). Neutrophil degranulation levels were corrected against the total protein concentration to rule out possible errors in counting cultured neutrophils in each condition. The supernatant’s total protein concentration was determined using the Bradford method.

### 2.6. Cytokine Assessment

Pro- and anti-inflammatory cytokine levels were measured in the cell culture supernatants using the Cytokine Human Magnetic 30-Plex Panel for Luminex™ Platform kit (#LHC6003M; Invitrogen, Carlsbad, CA, USA) following the manufacturer’s recommended instructions. The following cytokines were analyzed: fractalkine, GM-CSF, IFNγ, IL-1β, IL-4, IL-6, IL-7, IL-8, IL-10, IL-12p70, IL-17, IL-21, IL-23, ITAC, MIP-1α, MIP-1β, MIP-3α, and TNF-alpha.

TNF-alpha levels were confirmed by ELISA, using the commercial Human TNF-alpha DuoSet ELISA kit (#DY210; Bio-techne, Minneapolis, MN, USA) according to the manufacturer’s. Briefly, 96-well plates were coated with the anti-TNF-α capture antibody (for 24 h at room temperature). Plates were then washed three times with a solution of 0.05% Tween-20^®^ in Phosphate-Buffered Saline (PBS) (washing solution) (Sigma-Aldrich, Madrid, Spain) to remove any remaining unbound antibodies. After washing, wells were blocked with 1% bovine serum albumin (BSA) resuspended in PBS for 1 h at room temperature. For the assay, the samples (previously diluted 1:100) and the standard curve points (0–2000 pg/mL TNF-α) were added to the plate wells and incubated at room temperature for 2 h. Subsequently, 3 washes were performed, and the biotinylated detection antibody was added and incubated at room temperature for 2 h. The wells were washed 3 times with a washing solution, and streptavidin–HRP was added and incubated at room temperature for 20 min. After washing 3 times with the washing solution, HRP substrate was added to the wells and incubated at room temperature for 20 min. The colorimetric reaction was stopped by adding a stop solution (2N H_2_SO_4_). Absorbance was determined at 450 nm with the Spectramax Plus 384 (Molecular Devices, San Jose, CA, USA).

### 2.7. Statistical Analysis

Demographic and clinical data are expressed as median and range. The Shapiro–Wilk normality test was used to assess normality. Data following the normal distribution were analyzed using the ANOVA; otherwise, the Kruskal–Wallis non-parametric test was used. Multiple hypothesis testing was performed (Holm–Sidak and Dunn’s multiple comparisons tests) to identify pairwise differences among groups. The chi-square test was applied for proportion comparisons. Two-tailed *p* < 0.05 was considered statistically significant. Statistical analyses were performed using GraphPad Prism 9.0 software (GraphPad, La Jolla, CA, USA).

## 3. Results

### 3.1. Demographic and Clinical Data

Demographic and clinical data of individuals participating in this study are shown in Table 1. All participants were of pediatric age at the time of blood extraction. Patients were categorized according to their AAT phenotype (MZ, SZ, and ZZ). A control group of healthy volunteers with the MM phenotype was also included in this study. No significant differences in age (*p* = 0.06), gender (*p* = 0.69), or BMI (*p* = 0.39) were observed. All subjects included in this study were clinically healthy according to their physical, pulmonary, and liver tests. No significant differences were observed in pulmonary function tests or liver enzyme levels between the groups. As expected, significant differences between groups were observed in the AAT levels (*p* < 0.0001) (Table 1).

### 3.2. Degranulation Is Augmented in the Neutrophils of AATD Patients

Several markers of neutrophil activation and degranulation were analyzed to assess neutrophil activation.

**Figure 1:**
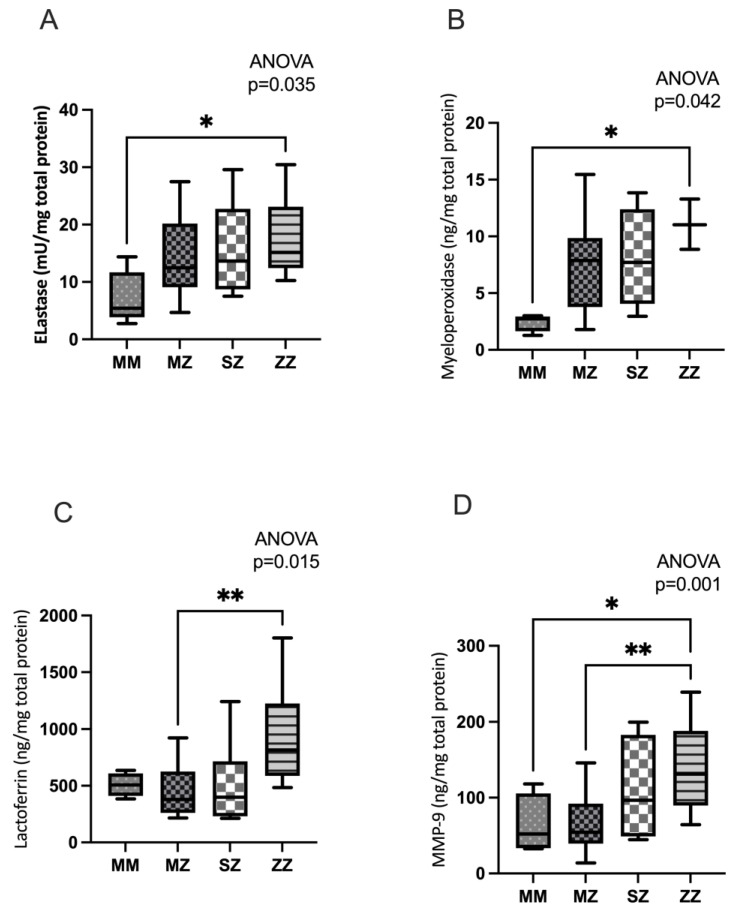
Neutrophils from ZZ AATD asymptomatic children show increased degranulation. Neutrophils were incubated under hypoxia for 4 h, primed with TNF-α (20 ng/mL for 30 min), and activated with fMLP (100 nM for 10 min). The presence of granule proteins was measured in the supernatants. (**A**) Elastase activity in the supernatants of AATD patients and healthy volunteers; (**B**) myeloperoxidase levels in the supernatants of AATD patients and healthy volunteers; (**C**) lactoferrin levels in the supernatants of AATD patients and healthy volunteers; (**D**) matrix metalloproteinase-9 (MMP-9) levels in the supernatants of AATD patients and healthy volunteers. * *p* < 0.05; ** *p* < 0.01.

Significant differences in NE activity were observed between the groups (*p* = 0.035) (Figure 1A). Multiple analysis testing revealed a significant increase in elastase activity in ZZ patients with respect to the control group (*p* = 0.025). Patients in the MZ and SZ groups showed higher levels than those in the control group, although the differences were not statistically significant (*p* = 0.108 and *p* = 0.4253, respectively). No statistically significant differences were observed when the patient groups were compared to each other.

Significant differences in MPO release were observed between groups (*p* = 0.042) (Figure 1B). Compared to the control group, ZZ patients showed statistically significantly elevated levels of MPO release (*p* = 0.030). MZ and ZZ patients showed higher levels of MPO than those in the control group, although the differences did not reach statistical significance (*p* = 0.2678 and *p* = 0.2916, respectively). When the patient groups were compared to each other, no statistically significant differences were observed.

Significant differences in lactoferrin release between groups (*p* = 0.015) were observed (Figure 1C). Multiple analysis testing showed a significant increase in lactoferrin levels in ZZ patients with respect to the MZ group (*p* = 0.007). In contrast, no statistically significant differences were observed when the patient groups were compared to each other.

Significant differences were observed in MMP-9 release between groups (*p* = 0.001). Multiple analysis testing showed a significant increase in MMP-9 in ZZ patients compared to the MM and MZ individuals (*p* = 0.044 and *p* = 0.001, respectively). No statistically significant differences were observed when patients were compared to each other.

### 3.3. Cytokine Profile

To assess how changes in cytokine profiles in AATD patients contribute to understanding the clinical phenotype and disease progression, a panel of pro- and anti-inflammatory cytokines was analyzed.

**Figure 2:**
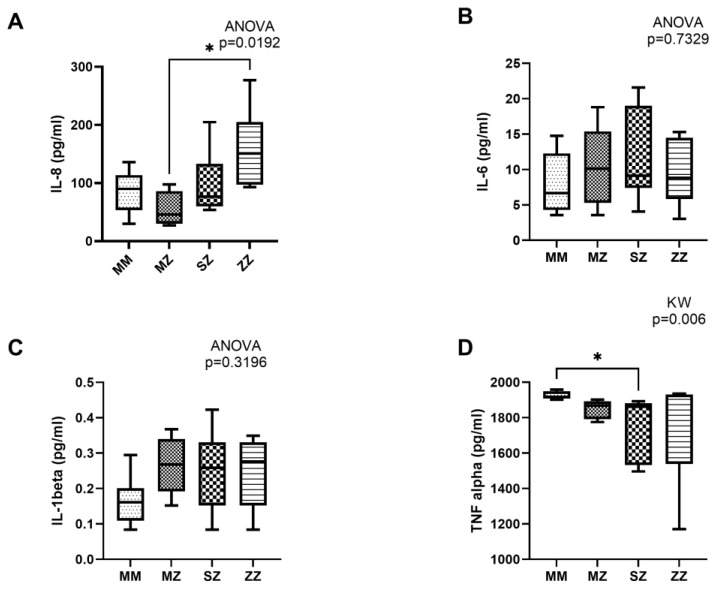
Neutrophil pro-inflammatory cytokine profile of control individuals and AATD patients. Neutrophils were incubated under hypoxia for 4 h, primed with TNF-α (20 ng/mL for 30 min), and activated with fMLP (100 nM for 10 min). Cytokine concentration was measured in the tissue culture supernatants. (**A**) Interleukin-8 (IL-8) concentration in the supernatants of AATD patients and healthy volunteers; (**B**) interleukin-6 (IL-6) concentration in the supernatants of AATD patients and healthy volunteers; (**C**) interleukin-1 beta (IL-1 beta) concentration in the supernatants of AATD patients and healthy volunteers; (**D**) tumor necrosis factor alpha (TNF-alpha) concentration in the supernatants of AATD patients and healthy volunteers. * *p* < 0.05.

Concerning pro-inflammatory cytokines, a significant increase in IL-8 (*p* = 0.019) was observed between the different groups (Figure 2A). Our results indicate a higher production of IL-8 in ZZ patients. However, significant differences were only observed when compared with the MZ group (*p* = 0.017), and no statistically significant differences were observed when compared with the rest of the groups (MM vs. ZZ, *p* = 0.269; SZ vs. ZZ, *p* = 0.2921). Similarly, a non-significant increase in IL-1 beta levels (*p* = 0.3196) was observed in AATD patients with respect to the control group (Figure 2C), with no significant differences in IL-6 production (*p* = 0.7329) (Figure 2B). A significant decrease was observed in TNF-alpha production between groups (*p* = 0.006). Multiple analysis testing showed statistically significant differences between MM and SZ (*p* = 0.012), and no statistically significant differences were observed between the rest of the groups (MM vs. MZ, *p* = 0.060; MM vs. ZZ, *p* > 0.999; MZ vs. ZZ, *p* = 0.644; SZ vs. ZZ, *p* = 0.2767) (Figure 2D).

**Figure 3:**
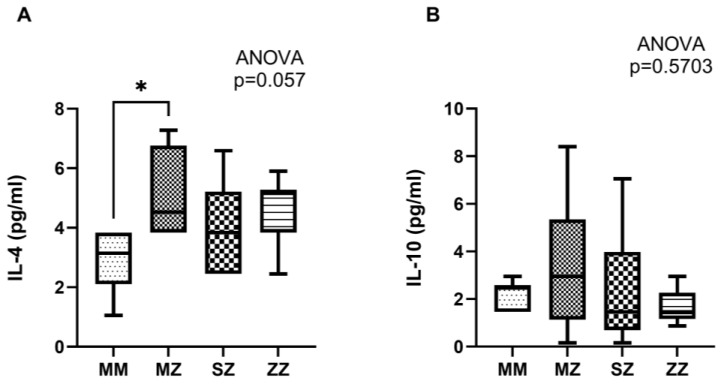
Neutrophil anti-inflammatory cytokine profile of control individuals and AATD patients. Neutrophils were incubated under hypoxia for 4 h, primed with TNF-α (20 ng/mL for 30 min), and activated with fMLP (100 nM for 10 min). Cytokine concentration was measured in the tissue culture supernatants. (**A**) Interleukin-4 (IL-4) concentration in the supernatants of AATD patients and healthy volunteers; (**B**) interleukin-10 (IL-10) concentration in the supernatants of AATD patients and healthy volunteers. * *p* < 0.05.

Regarding anti-inflammatory cytokines, a non-significant increase in IL-4 production was observed in the patient groups compared to the control group (*p* = 0.057) (Figure 3A). In contrast, no significant changes were observed in IL-10 production (*p* = 0.5703) (Figure 3B).

The remaining cytokines analyzed were below the detection limit of the technique used.

## 4. Discussion

Numerous studies have shown that neutrophils have a role in the pathophysiology of several inflammatory pulmonary illnesses associated with tissue hypoxia, including COPD [9], bronchiectasis [6], cystic fibrosis [10], neutrophilic asthma [6], and AATD [11,12]. Neutrophils’ level of activation has an impact on their capacity to damage nearby tissues. Excessive or unchecked neutrophil activation results in their degranulation and the release of proteolytic enzymes, whose accumulation in the extracellular space causes significant damage to nearby tissues [6,13]. It is now understood that neutrophils have the capacity to generate a range of mediators that have a significant impact on airways. These include proteases like matrix metalloproteinases, which can degrade the extracellular matrix (e.g., MMP-9, collagenase, and elastase). Through the enzyme myeloperoxidase, which catalyzes the creation of hypochlorous acid (HOCl), neutrophils are able to release harmful oxygen radicals. Neutrophils are also a source of pro-inflammatory cytokines such as TNF-α, interleukin-1, IL-6, IL-8, and Leukotriene B4 [6,13].

On the other hand, activated neutrophils consume more O_2_, resulting in local hypoxia, ROS production, and oxidative damage in the affected tissues [10,14,15,16]. However, the precise mechanisms by which neutrophils mediate tissue injury under hypoxic environments still need to be discovered [6].

Neutrophils have evolved cellular and molecular mechanisms that allow them to function effectively at low oxygen levels. Under physiological conditions, circulating neutrophils are exposed to a wide range of oxygen availability, from a pO_2_ of 13 kPa in the main arteries to 3 kPa in capillaries and venules (physiological hypoxia). Under homeostatic conditions, neutrophils are short half-life cells that easily undergo apoptosis to avoid degranulation and release of proteinases and consequent cell damage [13]. However, under hypoxic conditions, neutrophil survival is significantly prolonged, which is thought to delay the resolution of inflammation and promote tissue damage, potentially through the release of proteases and ROS [13,17,18]. Importantly, several studies have shown that the number of neutrophils in the lungs of AATD patients is significantly higher than those of healthy individuals, which could contribute to increased proteolytic and inflammation-hypoxia activity and the development of COPD (emphysema) observed in these patients [3].

However, previous studies on AATD neutrophils have been performed under atmospheric oxygen conditions (21% O_2_), which may not reflect physiological or pathological conditions [7,8,13]. In our study, increased neutrophil degradation in AATD patients was observed in hypoxia. Neutrophils from AATD patients, incubated under hypoxia for 4 h and stimulated with TNF-alpha and fMLP, showed significantly increased release of active NE (azurophil granules; *p* = 0.035) (Figure 1A), MPO (azurophil granules; *p* = 0.042) (Figure 1B), lactoferrin (specific granules; *p* = 0.015) (Figure 1C), and MMP-9 (gelatinase granules; *p* = 0.001) (Figure 1D) compared to control individuals (MM). When patients were compared to each other, a trend associated with phenotypic risk (ZZ > SZ > MZ) was observed that did not reach statistical significance, mainly as a result of the low sample size (see the limitations of this study below). In all cases, neutrophils from ZZ-AATD patients with a higher phenotypic risk of developing lung and liver disease showed, under hypoxic conditions, a significantly increased degranulation compared to the control group, except in the case of lactoferrin, where an increase associated with phenotype risk that does not reach statistical significance is observed.

The relationship between hypoxia and the regulation of neutrophil degranulation has been shown previously in neutrophils isolated from healthy volunteers incubated under hypoxic conditions [6]. Previous studies demonstrated that neutrophils isolated from healthy volunteers after hypoxemia showed increased NE release [13,19]. A later study revealed that neutrophils from healthy volunteers exposed to ex vivo hypoxia (3 kPA for 1–4 h) released elevated levels of NE [13,20]. A recent study has shown that neutrophils isolated from healthy volunteers cultured under hypoxic conditions (0.8% O_2_, 3 kPa for 4 h) released significantly higher levels of NE, MPO, lactoferrin, and MMP-9, indicating increased neutrophil degranulation. The same study observed that the supernatant from neutrophils cultured under hypoxic conditions induced airway epithelial cell death. This situation did not occur when cells were cultured with the supernatant from neutrophils cultured under normoxia. Subsequently, these researchers observed that co-incubation with AAT significantly reduced cell death, suggesting that NE was involved in cell damage [9]. Finally, a previous study has shown that neutrophils isolated from AATD patients with decreased lung function (FEV1 < 80%), incubated under atmospheric O_2_ conditions (21% O_2_) and stimulated with TNF-alpha and fMLP, showed a greater release of MPO than healthy individuals [21]. Our results agree with those previously published in which hypoxia was shown to induce the degranulation of neutrophils isolated from healthy volunteers. The increased production and release of neutrophilic proteases into the cellular environment suggests an increased ability to damage surrounding tissues [15]. However, this aspect has not been explored in the present study.

In our study, we have also evaluated the role of hypoxia in the production of a group of pro-inflammatory (IL-6, IL-8, IL-1 beta, and TNF-alpha) and anti-inflammatory (IL-4 and IL-10) cytokines to determine if hypoxia induces significant changes in cytokine production that could contribute to improving our understanding of the clinical phenotype and progression of the disease. Our results show a statistically significant increase in IL-8 (*p* = 0.019) and a trend to increase in IL-1 beta (*p* = 0.3196). Increased IL-8 in AATD can result in excessive accumulation of neutrophils in the lungs, which increases the release of elastases and other inflammatory mediators that contribute to alveolar damage and the development of emphysema [3]. IL-1β is involved in acute and chronic inflammatory processes. Its elevation in AATD patients suggests a continuous activation of inflammation, which may accelerate lung tissue degradation and exacerbate AATD-associated COPD [3]. Our results show no significant changes in the levels of IL-6 (*p* = 0.7329). This pro-inflammatory cytokine plays a very important role in the immune response and the regulation of systemic inflammation [3]. In this case, we would expect an increase in IL-6 levels that could indicate a chronic inflammatory response that would contribute to lung and liver damage and could be involved in the development of liver fibrosis observed in some patients. In our case, the unchanging levels in patients with AATD could indicate a different regulatory mechanism or inflammatory response stage than IL-8 and IL-1β. IL-6 also has roles in the immune response and in promoting inflammation. Still, its stability suggests that other factors may be more directly involved in these patients’ active pathogenesis of AATD.

Our results also show decreased TNF-alpha, a pro-inflammatory cytokine with complex roles in inflammation and apoptosis [3]. Interestingly, in our study, this cytokine decreases under hypoxia conditions in patients with AATD. Although we do not have an explanation for this decrease, the unexpected reduction in TNF-alpha in AATD may reflect an attempt by the body to modulate excessive inflammation and may also imply a failure of effective immune response against infections. Several studies have shown that hypoxia can reduce the release of TNF-alpha. Research indicates that chronic hypoxia can inhibit the up-regulation of TNF-alpha-induced monocyte chemoattractant protein-1 (MCP-1) expression in human proximal renal tubular cells, suggesting that hypoxia has a regulatory effect on inflammatory responses by decreasing TNF-alpha activity [22]. Another study found that hypoxia modulates the immune response by reducing the secretion of TNF-alpha, leading to lower NF-κB signaling and MCP-1 secretion in human adipocytes, further supporting the anti-inflammatory role of hypoxia in certain conditions [23]. These findings emphasize hypoxia’s potential to reduce inflammation by inhibiting TNF-alpha-related pathways.

Concerning anti-inflammatory cytokines, our results indicate a decrease in IL-10 and an increase in IL-4. IL-10 is an anti-inflammatory cytokine crucial in limiting the inflammatory response and preventing excessive tissue damage. Reducing IL-10 AATD may exacerbate inflammation and tissue damage by not adequately counteracting pro-inflammatory cytokines. This could contribute to the rapid progression of lung and liver disease in these patients. On the other hand, IL-4 is a cytokine associated with humoral immune response and Th2 cell activation. An increase in IL-4 in AATD may indicate a shift toward a Th2-type immune response, which is less effective in dealing with infections and more prone to chronic inflammation and fibrosis. This shift may aggravate lung and liver problems, promoting a chronic inflammatory environment.

Overall, our results indicate that the cytokine profile in AATD patients fits within an unbalanced inflammatory environment. Increased pro-inflammatory cytokines such as IL-8 and IL-1β suggest chronic active inflammation contributing to tissue damage. Decreased TNF-alpha and IL-10 indicate inadequate regulation of inflammation, which may result in ongoing damage and a compromised immune response. In contrast, increased IL-4 signals a possible shift toward a Th2-type immune response, which may not be beneficial in the context of AATD, as it may promote fibrosis and exacerbate inflammation.

Previous studies have shown that the involvement of cytokines in AATD is complex and involves both pro-inflammatory and anti-inflammatory cytokines. In AATD, the inflammatory response is increased due to the lack of AAT’s protective effects [24]. Several studies have highlighted the elevated levels of pro-inflammatory cytokines in AATD patients [3]. Elevated IL-8 levels in AATD patients have been associated with increased neutrophil infiltration and subsequent lung tissue damage. The recruitment of neutrophils exacerbates the release of NE, fostering the cycle of inflammation and tissue destruction [25,26,27]. IL-1 beta shows increased levels in AATD patients, which contributes to activating inflammatory pathways and promoting cytokine cascades. IL-1β induces the expression of adhesion molecules and other cytokines, perpetuating the inflammatory state and contributing to tissue damage [28].

Several studies have shown increased TNF-alpha production in AATD, showing that lack of AAT results in increased TNF-alpha production and augmented neutrophil degranulation [3]. Our results do not agree with those of other studies. Contrary to expected, a reduction in TNF-alpha production is observed, which might reflect a compensatory mechanism that attempts to control chronic inflammation. However, the decreased TNF-alpha may also indicate an impaired immune response, unable to regulate the tissue damage observed in AATD [22,23]. So far, we do not have an explanation for these results, and they deserve further research.

A major limitation of our study is the low number of patients with SZ and ZZ phenotypes included, which is not surprising given the low prevalence of these phenotypes. This may be why some of the results (particularly those related to the study of cytokines) do not reach statistical significance, even though a clear trend is observed in many cases. However, the significant differences observed in some of the studied degranulation markers indicate that their results should remain relatively constant after an increase in the sample size of the study groups mentioned above. For the same reason, possible sex/gender differences were not analyzed, limiting the results’ generalizability. One of the strengths of our study is that it has been performed on isolated neutrophils from clinically healthy AATD children. Respiratory function (assessed by spirometry performed in this study and previous X-ray and CT scans that revealed no signs of lung damage) and liver enzyme makers were normal in all patients included in this study. Since (at least in theory) children do not consume tobacco or alcohol, the bias introduced by these factors can be avoided, so the results obtained can be attributable to AATD and not to the influence of confounding factors.

This is the first time that the role of hypoxia in neutrophil degranulation and cytokine production in AATD patients has been investigated. Since hypoxia is a crucial component of the pathophysiology of many illnesses, including COPD, heart failure, sleep apnea syndrome, and cancer, basic research into hypoxia can uncover processes that could be used to develop new therapeutic strategies [6,15].

In summary, the main contribution of this study to current knowledge on AATD neutrophil degranulation relies upon the effects of hypoxia and cytokine production, which confers a much more aggressive neutrophilic phenotype that could be related to the increased lung and liver damage observed in these patients. Our results show that the ability of ZZ-AAT neutrophils to release the protease content of their granules to the extracellular matrix is increased compared to MM-healthy individuals, suggesting that ZZ-AATD neutrophils might have a greater capacity to damage adjacent tissues. Finally, we have shown an imbalance between pro-inflammatory and anti-inflammatory cytokines, which may contribute to chronic inflammation and tissue damage. Therefore, it is important to understand the role of cytokines in AATD since it may have therapeutic implications. Targeting specific cytokines to restore balance could mitigate inflammation and tissue damage [29].

## Figures and Tables

**Table 1:** Demographics and clinical characteristics of AATD patients and control individuals.

Variable	MM (n = 7)	MZ (n = 31)	SZ (n = 8)	ZZ (n = 15)	*p*-Value
**Age (yr; range)**	13 (3–15)	8 (1–14)	10 (8–12)	7 (1–13)	0.06
**Gender** **Male/Female (%)**	43/57	52/48	75/25	54/46	0.69
**AAT (mg/dL)** **(Norm: 90–200)**	130 (110–155)	84 (67–141)	55 (52–79)	23 (19–38)	**<0.0001**
**BMI (Kg/m^2^)**	19 (15–22)	17 (12–22)	17 (15–19)	18 (14–26)	0.39
**FEV_1_** **(Norm: ≥80% predicted)**	99 (71–112)	101 (80–106)	108 (98–117)	99 (88–127)	0.64
**FVC** **(Norm: ≥80% predicted)**	100 (73–116)	99 (81–126)	99 (88–109)	95 (79–133)	0.86
**FEV_1_/FVC** **(Norm: >80% predicted)**	96 (72–98)	92 (79–96)	94 (84–98)	91 (82–96)	0.75
**AST (U/L)** **(Norm: 1–37)**	25 (19–43)	31 (19–40)	34 (20–36)	35 (29–41)	0.13
**ALT (U/L)** **(Norm: 1–37)**	19 (8–52)	19 (12–50)	20 (18–28)	33 (21–43)	0.30
**GGT (U/L)** **(Norm: 1–55)**	17 (10–38)	15 (12–22)	17 (13–22)	19 (18–20)	0.40

Data are presented as median and range (minimum and maximum values). Abbreviations are as follows: AAT, Alpha-1 antitrypsin; BMI, body mass index; FEV_1_, forced expiratory volume in 1 s; FVC, forced vital capacity; AST, aspartate aminotransferase; ALT, alanine aminotransferase; and GGT, gamma-glutamyl-transferase. Normality (Norm) values are indicated. *p*-values lower than 0.05 were statistically significant (labeled in bold).

## Data Availability

Data are unavailable due to privacy and ethical restrictions.

## Supplementary Materials

The following supporting information can be downloaded at: https://www.mdpi.com/article/10.3390/antiox13091071/s1, Figure S1: Purity of the neutrophil culture after isolation by negative immunomagnetic selection determined by flow cytometry.
