# Supplementary material for: Impact of Hypoxia on Neutrophil Degranulation and Inflammatory Response in Alpha-1 Antitrypsin Deficiency Patients
Source: Antioxidants (Basel). 2024 Sep 2;13(9):1071. doi: 10.3390/antiox13091071 (PMC11428696; doi:10.3390/antiox13091071)
Supplement: Supplementary file 1 [file antioxidants-13-01071-s001.zip › antioxidants-3140988-supplementary.pdf]

Supplementary materials S1.

The purity of isolated neutrophils was evaluated by flow cytometry (LSR Fortessa X-20 (BD Biosciences)) using a monoclonal anti-CD45 antibody (PE anti-human CD45 Antibody; #304007; BioLegend) used as a total leukocyte marker, with higher expression in lymphocytes, and another anti-CD16 monoclonal antibody (APC anti-human CD16 Antibody; #360705; BioLegend), specific for neutrophils. The isolated sample showed a purity of 96.94% of neutrophils corresponding to the individual CD16-positive cells (Figure 1).

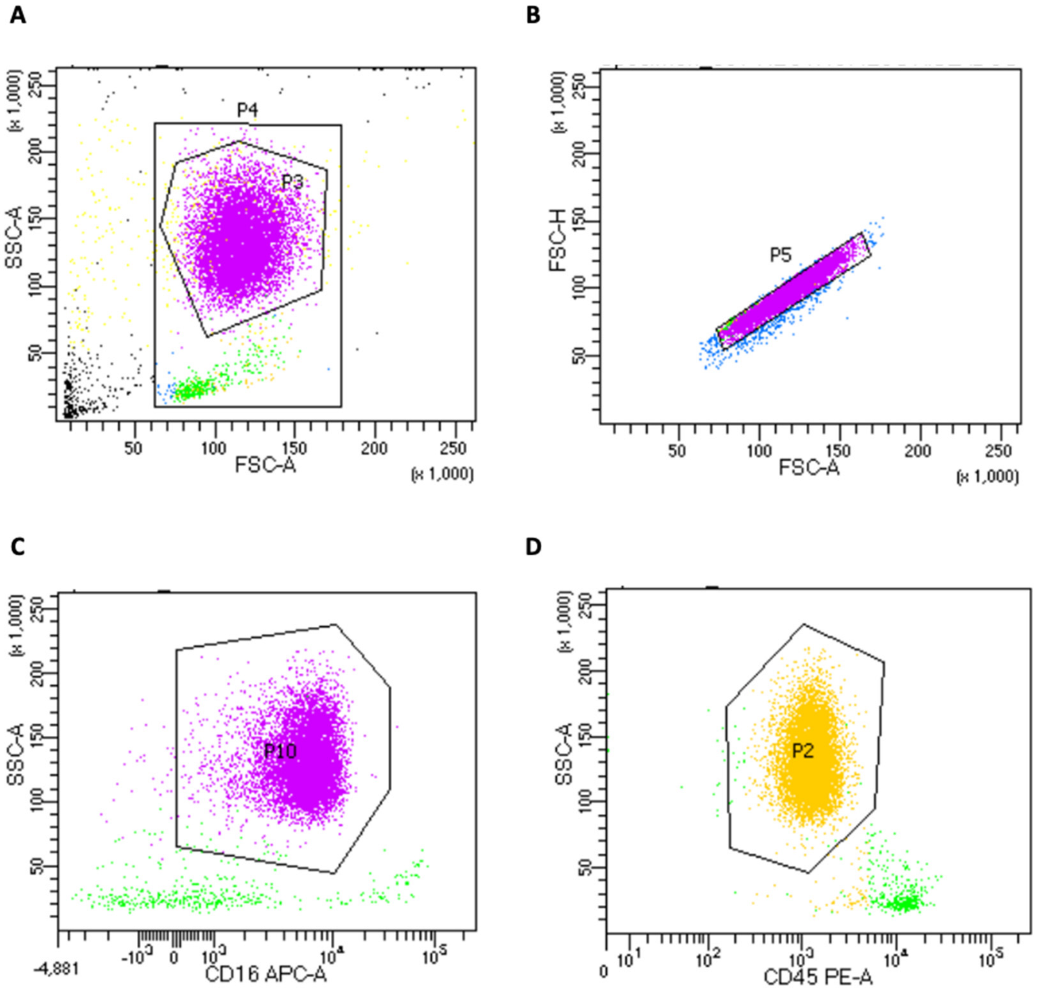

Figure S1. Purity of the neutrophil culture after isolation by negative immunomagnetic selection determined by flow cytometry. A) Shows the total events in the sample, B) exclusion of cell aggregates, C) cells in the sample labeled with the APC-conjugated anti-CD16 antibody (neutrophil marker), D) cells in the sample labeled with the PE-conjugated anti-CD45 antibody (leukocyte marker).
